# Molecular Mechanisms of Possible Action of Phenolic Compounds in COVID-19 Protection and Prevention

**DOI:** 10.3390/ijms222212385

**Published:** 2021-11-17

**Authors:** Nikola Gligorijevic, Mirjana Radomirovic, Olgica Nedic, Marija Stojadinovic, Urmila Khulal, Dragana Stanic-Vucinic, Tanja Cirkovic Velickovic

**Affiliations:** 1 Institute for the Application of Nuclear Energy, Department for Metabolism, University of Belgrade, Banatska 31b, 11080 Belgrade, Serbia; nikolag@inep.co.rs (N.G.); olgica@inep.co.rs (O.N.); 2 Center of Excellence for Molecular Food Sciences, Department of Biochemistry, Faculty of Chemistry, University of Belgrade, Studentski trg 12-16, 11000 Belgrade, Serbia; radomirovicmirjana@chem.bg.ac.rs (M.R.); mstojadinovic@chem.bg.ac.rs (M.S.); dstanic@chem.bg.ac.rs (D.S.-V.); 3 Faculty of Bioscience Engineering, Ghent University, 9000 Ghent, Belgium; Urmila.Khulal@ghent.ac.kr; 4 Global Campus, Ghent University, Yeonsu-gu, Incheon 21985, Korea; 5 Serbian Academy of Sciences and Arts, Knez Mihailova 35, 11000 Belgrade, Serbia

**Keywords:** SARS-CoV-2, polyphenols, antiviral effects, antiviral targets

## Abstract

The worldwide outbreak of COVID-19 was caused by a pathogenic virus called Severe Acute Respiratory Syndrome Coronavirus-2 (SARS-CoV-2). Therapies against SARS-CoV-2 target the virus or human cells or the immune system. However, therapies based on specific antibodies, such as vaccines and monoclonal antibodies, may become inefficient enough when the virus changes its antigenicity due to mutations. Polyphenols are the major class of bioactive compounds in nature, exerting diverse health effects based on their direct antioxidant activity and their effects in the modulation of intracellular signaling. There are currently numerous clinical trials investigating the effects of polyphenols in prophylaxis and the treatment of COVID-19, from symptomatic, via moderate and severe COVID-19 treatment, to anti-fibrotic treatment in discharged COVID-19 patients. Antiviral activities of polyphenols and their impact on immune system modulation could serve as a solid basis for developing polyphenol-based natural approaches for preventing and treating COVID-19.

## 1. Introduction

The worldwide outbreak of COVID-19 was caused by a pathogenic virus called Severe Acute Respiratory Syndrome Coronavirus-2 (SARS-CoV-2), belonging to the β-coronaviruses lineage B. At the beginning of the pandemic, respiratory symptoms led to the assumption that COVID-19 exclusively affects the respiratory system, but later on, growing evidence demonstrated also SARS-CoV-2-mediated extra-respiratory manifestations and complications, such as cardiac, gastrointestinal, hepatic, renal, neurological, olfactory, ocular, cutaneous, hematological, and reproductive [1,2,3], clearly demonstrating that COVID-19 is a multisystem disease. The virus uses a surface glycoprotein, called a ‘spike’ protein (S protein), to bind to angiotensin-converting enzyme 2 (ACE2) and enter the host cell. ACE2 has a role in the regulation of vasoconstriction and blood pressure, and although it is most abundant on type II alveolar cells, several studies have shown that ACE2 is expressed not only in lung tissues but also in extra-pulmonary organs such as the heart, kidney, liver, colon, esophagus, brain, gallbladder and testis, explaining why SARS-CoV-2 may also affect extra-pulmonary organs [4].

Therapies against SARS-CoV-2 target the virus or human cells or the immune system. The key SARS-CoV-2 targets comprise three non-structural proteins (3CL^pro^, PL^pro^, and RdRp) and a structural protein (S protein), which are responsible for replication, transcription, and host cell recognition [5]. However, on the one hand, therapies based on specific antibodies, such as vaccines and monoclonal antibodies, may become inefficient enough when the virus changes its antigenicity due to mutations. On the other hand, many serious adverse effects have been reported during the application of pharmacological treatments [6]. Therefore, novel strategies with improved efficiency and safety are still needed. As nature offers a tremendous treasury of different chemical compounds with anti-viral and health-promoting activity at the same time, an increasing number of studies are dedicated to uncovering the potential of natural products, such as polyphenols, to combat COVID-19 [7,8,9,10,11].

Polyphenols are the major class of bioactive compounds in nature, exerting diverse health effects based on their direct antioxidant activity and their effects in the modulation of intracellular signaling [12,13]. Antiviral activities of polyphenols and their impact on immune system modulation could serve as a solid basis for developing polyphenol-based natural approaches for preventing and treating COVID-19 [14]. Besides antiviral activities and immune system regulation, the pleiotropic and multi-organ health-promoting effects of polyphenols [15,16] make them even more beneficial to combat COVID-19, taking into account the multisystem complications of this disease. In addition, polyphenols are natural food components and thus safe even at high daily doses (gram quantities) [17], available from sustainable resources, inexpensive, and easy to isolate/purify, even from food industry waste.

Therefore, this review aims to present molecular mechanisms of possible action of polyphenols in protection and prevention against COVID-19, based on literature data obtained from in vitro, in vivo, and clinical studies, as well as to review the potential of polyphenol structures as inspiration for drugs under development to be used for COVID-19 prevention and treatment. Literature data for this review was collected using the Scopus database in the period from 2019 to 2021. Keywords used for the search were polyphenols, COVID-19, SARS-CoV-2, Spike protein, 3CL^pro^, M^pro^, and PL^pro^. Due to the high similarity between SARS-CoV and SARS-CoV-2 key protein targets, some in vitro effects of polyphenols previously reported for SARS-CoV are also presented.

## 2. Health Effects of Polyphenols Related to COVID-19

**Figure 1:**
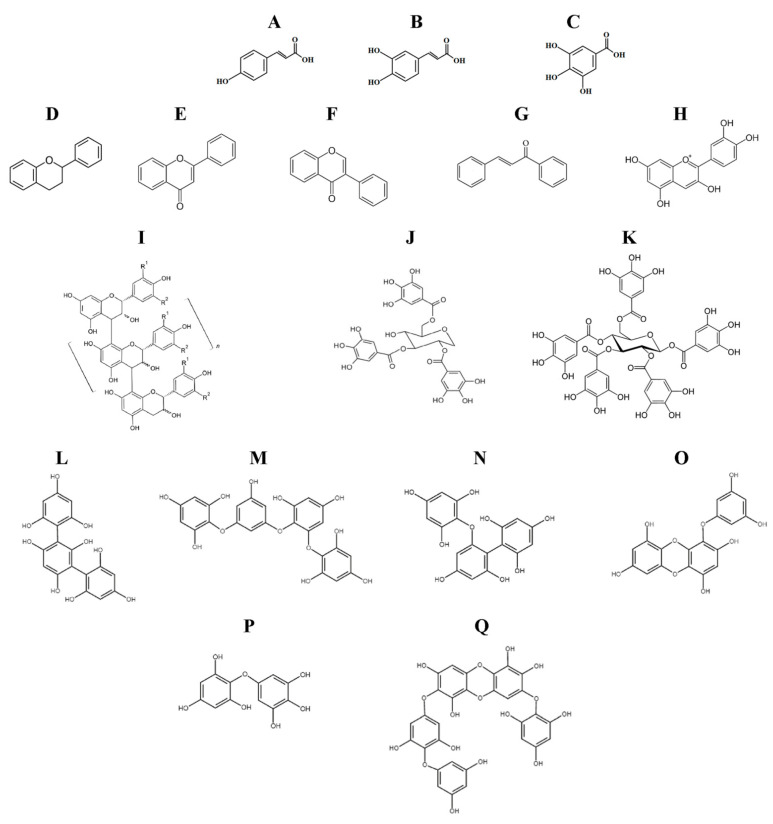
Structures of some phenolic acids and several classes of polyphenols. *p*-Coumaric acid (**A**), caffeic acid (**B**), gallic acid (**C**), flavan (**D**), flavone (**E**), isoflavone (**F**), chalcone (**G**), anthocyanidin (**H**), condensed tannin (**I**), gallotannin (**J**), ellagitannin (**K**), fucol (**L**), phloroethol (**M**), fucophloroethol (**N**), eckol (**O**), fuhalol (**P**), and carmalol (**Q**).

Polyphenols, the major class of bioactive compounds in nature, are plant secondary metabolites comprising compounds of diverse structures, molecular weights, and properties and are ubiquitously present in plant-based foods. According to the polyphenol definition of Quideau et al. [18], the term “polyphenol” should be used to define plant secondary metabolites derived exclusively from the shikimate-derived phenylpropanoid and/or the polyketide pathway(s), featuring more than one phenolic ring and being devoid of any nitrogen-based functional group in their most basic structural expression. Although all monophenolic structures, such as phenolic acids, and the naturally occurring derivatives, such as methyl phenyl ethers and *O*-phenyl glycosides, are excluded from this definition, due to their various biological activities resembling those of polyphenols, these compounds are studied in parallel with true polyphenols for their sanative health effects. The structures of some phenolic acids and polyphenol classes are presented on Figure 1.

A structural feature common to all plant phenolic compounds is the presence of an aromatic ring and at least one hydroxyl group. Compounds with more than one phenolic ring are known as polyphenol compounds. Therefore, to be precise, phenolic acids, with their structures of the C6-C1 (benzoic acid derivatives) or C6C3 (cinnamic acid derivatives) types, are not polyphenols. The most common dietary phenolic acids are caffeic acid, gallic acid, ferulic acid, chlorogenic acid, and *p*-coumaric acid [19].

Flavonoids are polyphenols with the C6-C3-C6 general backbone structure, where the two C6 units (Ring A and Ring B) of a phenolic nature are bridged by chromane ring (Ring C), and according to the hydroxylation pattern and variations in the chromane ring flavonoids are divided into flavans, flavanones, isoflavanones, flavones, isoflavones, anthocyanidins, chalcones, and flavonolignans [20].

Some polyphenols may undergo oligomerization to form higher molecular weight compounds, commonly known as “true” vegetable tannins. The condensed tannins are also known as proanthocyanidins, such as procyanidins, prodelphinidins, and profisetinidins, which are formed by the oligomerization of flavan-3-ol units such as (epi)catechin, epigallocatechin, and fisetinidol. Hydrolyzable tannins include the gallo- and ellagitannins (hydrolyzable tannins), which are derived from the metabolism of the shikimate-derived gallic acid that leads through esterification and phenolic oxidative coupling reactions to numerous (close to 1000) monomeric and oligomeric polyphenolic galloyl ester derivatives of sugar-type polyols, mainly D-glucose [18]. The phlorotannins are synthesized by red-brown algae and are structurally analogous to tannins from terrestrial plants. They are comprised of polymeric chains of base phloroglucinol (1,3,5-trihydroxybenzene) residues connected via C-C and/or C-O-C couplings and are classified as fucols (phenyl bond), phloroethols (ether bond), fucophloroethols (ether and phenyl bonds), eckols, fuhalols, and carmalols [21].

The effects of polyphenols on human health are based on phenolic–protein interactions, covalent and/or noncovalent (hydrophobic interactions, hydrogen bonding, van der Waals interactions, electrostatic interactions, steric repulsive forces, ion bridging, and dipole–dipole/charge–dipole interactions). At the molecular level, by interactions of polyphenols and their metabolites with functional proteins, such as intracellular enzymes, transcription factors (TFs), receptors and other functional proteins, via a multiple-target mechanism and in a complex interaction manner, polyphenols modulate regulation of cell events, signaling pathways, and metabolic processes [22].

The health benefits of polyphenols have been the focus of thousands of studies published in the last few decades. With their antiviral, antioxidant, anti-inflammatory and multi-organ protective effects, polyphenols are promising potent weapons to mitigate the major pathways involved in the pathogenesis of SARS-CoV-2, as well as to improve recovery of impaired multi-organ systems in post-COVID-19 patients.

Several recent reviews recognized and presented different aspects of potential use of polyphenols against COVID-19 by assessing the effects of polyphenols on COVID-19 drug targets [5]; the underlying mechanism of polyphenols antiviral and immune-regulation activities in COVID-19 [23]; the possibility to use polyphenols for the development of novel natural approaches against COVID-19 [6,24]; polyphenols potential in strengthening antioxidant defenses and upregulating the immune systems; and in prevention, replication, and spreading of the SARS-CoV-2 [25]. Similarly, the role of key medicinal/nutritional antioxidants, including polyphenols, in the antiviral immune response in COVID-19 [26], mechanism of polyphenol action in COVID-19 in comparison to other acute infectious diseases [27], potential of polyphenols in reducing COVID-19 cytokine storm [28,29], and potential of polyphenols in tuning of autophagy and ubiquitin-proteasome system pathways in COVID-19 [30] were reviewed. The use of tea polyphenols in prophylaxis and treatment of COVID-19 [31,32]; polyphenol potential for modulating the expression of host microRNAs that play role in SARS-CoV-2 infection [33]; and beneficial effects of polyphenols against the COVID-19-induced lung damage and associated systemic effects based on predictive, preventive, and personalized medicine (3PM) were also critically studied [34].

Although the total polyphenol intake for the general population is estimated to be 0.9 g per day [35], upon ingestion, only 5–10% of the total polyphenol intake is absorbed in the small intestine, while the remaining 90–95% may accumulate in the large intestinal lumen up to the millimolar range [36]. In the gastrointestinal tract (GIT), polyphenols are subjected to phase I/II metabolism and then carried out in gut and liver cells. In contrast, unabsorbed polyphenols are degraded by microflora in the colon and thereafter partly absorbed by the hepatic portal vein. From the liver, polyphenols and their metabolites enter systemic circulation and are absorbed by peripheral tissues where they exert their bioactivities [13]. Therefore, despite high daily intake, in peripheral tissues intact polyphenols reach very low concentrations, typically in the nanomolar range, presumably too low to have a direct impact on the antioxidant capacity [37]. Still, their metabolites, found in comparably greater amounts, may act as direct radical scavengers and mainly contribute to exerted bioeffects [13]. Consequently, future studies on the beneficial effects of polyphenols in COVID-19 should also take into account polyphenol bioavailability, the contribution of their metabolites in observed bioactivities, and their effective concentrations in target tissues. It is worth mentioning that appropriate combinations of different phenolics were shown to enhance the bioactivities of individual compounds, resulting in a positive synergistic/additive effect depending on the amounts of the phenolics [22]. Therefore, future studies should focus on testing these proved beneficial combinations of polyphenolics in the fight against COVID-19.

When administered orally, polyphenols should have unambiguous beneficial effects in preventing and treating COVID-19, at least in GIT. In fact, SARS-CoV-2 is frequently found in the stool, and gastrointestinal tissue samples of patients with COVID-19 [38], and SARS-CoV-2 also infects the GIT via ACE2 receptor highly expressed throughout the GIT [39]. This suggests that, while the respiratory tract is the primary site for SARS-CoV-2 infection, the fecal-oral route is an alternative route by which SARS-CoV-2 can spread [32]. On the other hand, GIT dysfunction in critically ill COVID-19 patients may be related to critical illness and associated systemic inflammation and treatments, independent of GIT infection [40]. Therefore, upon polyphenol ingestion, excessive concentrations (in millimolar range) of polyphenols and their metabolites along GIT could be of great importance in the fight against COVID-19 in situ, particularly in the cases of SARS-CoV-2 infection of GIT, prevention of fecal-oral transmission, and alleviation of GIT complications in critically ill patients. It is known that upon ingestion, polyphenols interact with proteins within the oral cavity, such as proline-rich proteins secreted in the saliva [41] or saliva mucins [42]. Moreover, since high expression of ACE2 receptor of SARS-CoV-2 was found on the epithelial cells of the oral mucosa, particularly on epithelial cells of the tongue, the oral cavity is considered as potential high risk for SARS-CoV-2 infection [43]. Therefore, upon ingestion, polyphenols could inhibit SARS-CoV-2 entry and its replication, thus reducing risk for SARS-CoV-2 infection by binding to viral and/or host proteins in the oral cavity.

There are currently numerous clinical trials (Table 1) investigating the effects of polyphenols in prophylaxis and the treatment of COVID-19 from symptomatic via moderate and severe COVID-19 treatment to anti-fibrotic treatment in discharged COVID-19 patients. In these clinical trials, polyphenols are assessed as pure polyphenols, pure polyphenols in combination with vitamins/minerals and/or other natural bioactive compounds or drugs, polyphenol-rich extracts, or polyphenol-rich extracts in combination with other bioactive components or drugs.

## 3. Overview of SARS-CoV-2 and Its Main Antiviral Protein Targets

**Figure 2:**
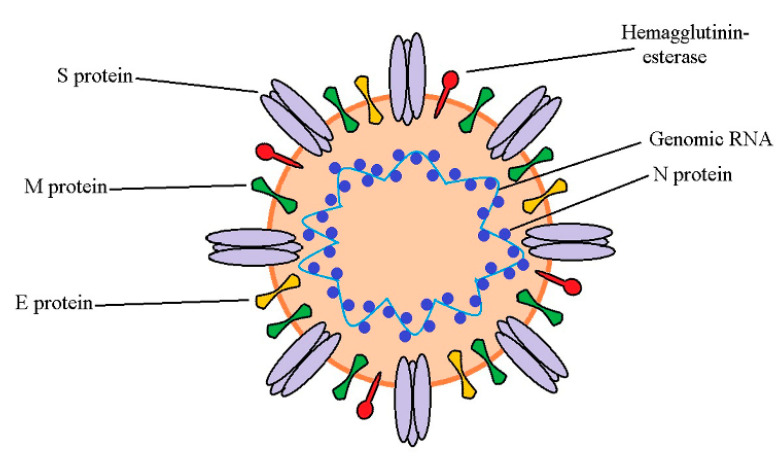
Structure of SARS-CoV-2 virus.

The virus responsible for the COVID-19 pandemic, SARS-CoV-2, is the seventh coronavirus that can infect humans. Like other coronaviruses, it has either a spherical or pleomorphic structure with a viral particle size of about 150–160 nm [27]. It contains positive single-stranded RNA. Structural proteins of this virus are envelope protein (E), nucleocapsid protein (N), membrane glycoprotein (M), and spike glycoprotein (S) [44]. Unlike other coronaviruses, SARS-CoV-2 has additional glycoprotein with acetylesterase and hemagglutination activity. SARS-CoV-2 genome organization is typical for coronaviruses, consisting of 5′ and 3′ untranslated regions, two open reading frames, ORF1a and ORF1b, and the following genes: S, N, M, and E. Two ORF regions code for 16 nonstructural proteins (nsp), namely nsp1-16 [45]. Between ORF1a and ORF1b regions is the location of −1 frameshift, and as a consequence, two polypeptides are coded, pp1a and pp1ab. These polypeptides are further processed by viral-encoded proteases, thus forming 16 nsp [46]. The overall structure of SARS-CoV-2 is shown in Figure 2.

Coronaviruses, including SARS-CoV-2, have a remarkable ability for interspecies transmission, which is, at least in part, attributed to various strategies they employ to infect target cells [47]. Spike glycoprotein (S protein), consisting of two subunits named S1 and S2, is the protein mainly responsible for coronavirus infectivity and host specificity. Similar to SARS-CoV, cell entry of SARS-CoV-2 is dependent on binding of S1 subunit to cellular angiotensin-converting enzyme 2 (ACE-2) receptor and S protein priming at S1/S2 and S2′ sites by host cell transmembrane protease, serine 2 (TMPRSS2), and cysteine proteases cathepsin B and L [48]. Unlike SARS-CoV, cell entry of SARS-CoV-2 is preactivated by proprotein convertase furin [49]. Upon cleavage, the S2 subunit mediates fusion of viral envelope, host cell membrane, and endosome. The activity of S2 and lower pH leads to the viral genome’s release into the cytoplasm [50]. At this point, the endoplasmic reticulum is utilized to produce double-membrane vesicles, which protect the viral genome and enable replication to occur. With the help of the host cell, the viral genome is translated to polypeptides which are then separated into structural and nonstructural proteins with the action of two viral proteases, papain-like protease (PLpro) and 3-chymotrypsin-like protease (3CL^pro^). As 3CL^pro^ cleaves most polypeptide sites, it is also known as the main protease or Mpro. Assembly of whole virions takes place in the endoplasmic reticulum and Golgi apparatus. Virions then leave the host cell by exocytosis and are ready to infect new cells [51]. Although S protein plays a crucial role in SARS-CoV-2 infectivity, other proteins are also of great importance for the structural integrity of the virus and its function. For instance, N protein is essential for the viral structure and its ability to bind viral RNA, making ribonucleoprotein complex, which is crucial for the replication of the virus. Both E and M proteins play a role in viral particles assembly, while E protein also serves as an ion channel [52].

Essential proteins involved in the virus replication cycle are often targets of antiviral drugs. An overview of major proteins of SARS-CoV-2 acting as antiviral targets will be given in the following subsection.

### 3.1. Primary Antiviral Protein Targets of SARS-CoV-2

#### 3.1.1. Spike (S) Protein

**Figure 3:**
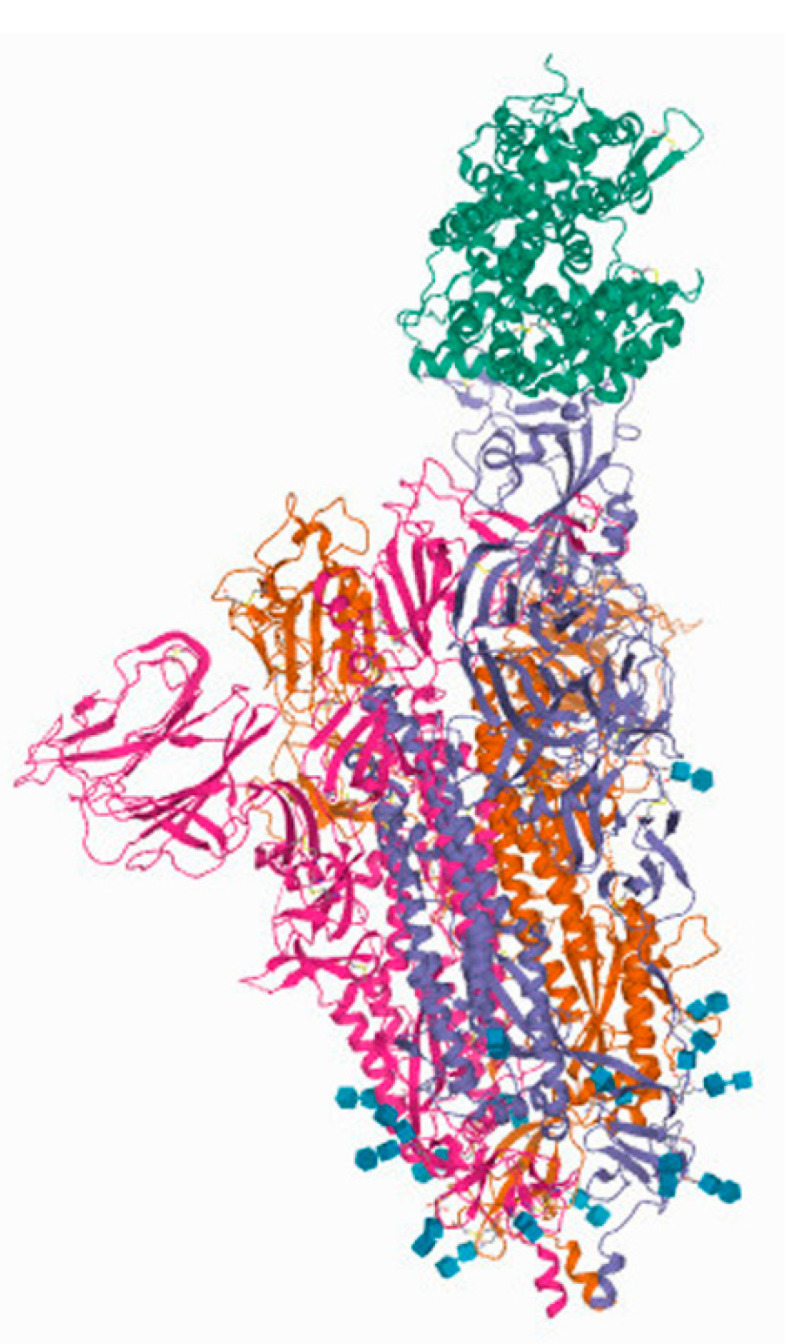
Structure of SARS-CoV-2/ACE 2 receptor complex. The upper, wider part is the S1 domain that interacts with the ACE2 receptor, colored in green, while the lower, narrower part is the S2 domain. Blue squares represent carbohydrates. Image obtained by Cong et al., 2021 [55], PDB entry: 7DF4.

When considering SARS-CoV-2 structural proteins, spike or S-protein is getting the most attention being the one responsible for the attachment of the virus to the host cell. S protein has a molecular mass of 180–200 kDa in a monomeric state, and it consists of two domains, S1 and S2. S1 domain, responsible for binding to ACE2 receptor, is composed of N-terminus domain and receptor-binding domain (RBD). S2 domain, responsible for viral fusion with the host cell membrane, consists of fusion peptide, heptapeptide repeat sequence 1 and 2 (HR 1 and 2), transmembrane domain (TM), and cytoplasm domain (CT). In the native state, S protein is a trimer. Its structure has been determined by cryo-electron microscopy. It was noted that it could exist in two forms, named closed and opened. Hinge-like movement of the RBD domain makes these two forms, with opened one being able to interact with ACE2 receptor [53,54]. The structure of the S protein interacting with the ACE2 receptor is shown in Figure 3.

Different strategies are being utilized to find the appropriate way to interrupt the proper function of S viral protein, including prevention of its binding to ACE2 receptor, inhibition of its fusion function, and inhibition of proteases responsible for its cleavage [53]. Antibodies, synthetic peptides, and other molecules, including polyphenols, are being tested for this application. Considering that RBD domains of SARS-CoV and SARS-CoV-2 have about 75% homology in amino acid sequence, antibodies and molecules that target this domain might not be equally effective for both viruses [56].

#### 3.1.2. CL^pro^/M^pro^

**Figure 4:**
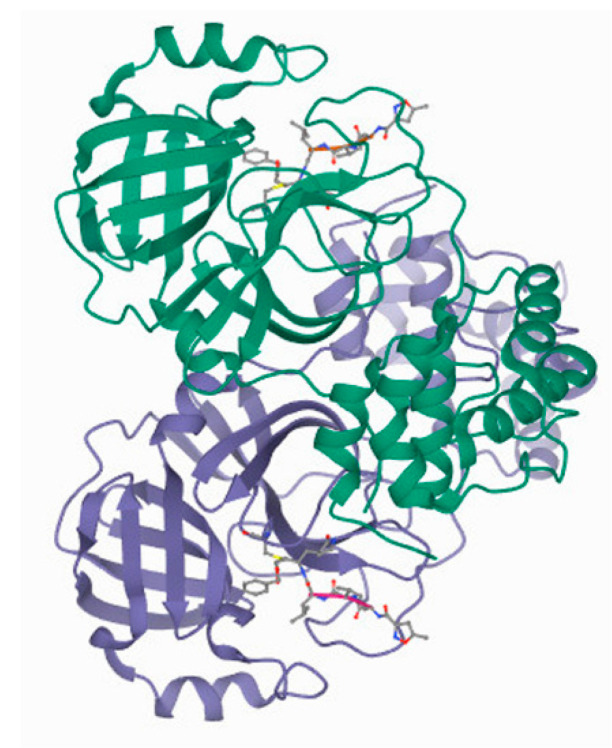
Crystal structure of SARS-CoV-2 3CL^pro^/M^pro^ bound to its inhibitor named N3. Inhibitors are located at the two substrate binding sites. Two protomers, A and B, are presented in green and purple. Image obtained by Jin et al., 2020 [57], PDB entry: 6LU7.

3CL^pro^/M^pro^, Figure 4, is a nonstructural protein of coronaviruses in general. This enzyme cleaves viral polyproteins, thus creating proteins required for virus replication and maturation. Two polypeptides named protomer A and B form a dimeric structure of 3CL^pro^. Each protomer is composed of three domains. Domains I and II have antiparallel β-barrel structure, while domain III has five α-helices. An extended loop region connects domains II and III. The location of Cys-His catalytic dyad of 3CL^pro^ and substrate binding site is in a cleft between domains I and II. These structural characteristics are similar to M^pro^ enzymes of other coronaviruses [57].

Inhibition of 3CL^pro^ prevents replication of the virus, which makes this protease an ideal therapeutic target. Amino acid sequence similarity of 3CL^pro^ from SARS-CoV-2 is about 96% compared to the one from SARS-CoV with a difference in only 12 out of 303 positions in the amino acid sequence. Crystal structures of SARS-CoV and SARS-CoV-2 main proteases showed that the active sites of these enzymes are conserved [58]. Considering there is high homology between the two proteases, it is plausible to speculate that effective inhibitors discovered for 3CL^pro^ from SARS-CoV would also be effective against the same protease from SARS-CoV-2.

#### 3.1.3. PL^pro^

**Figure 5:**
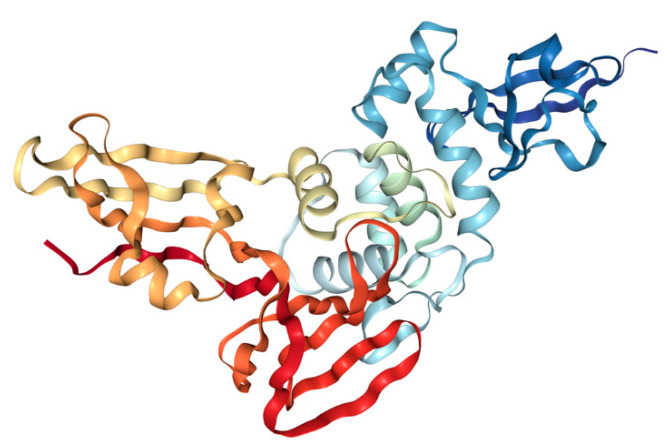
Crystal structure of PL^pro^ protease from SARS-CoV-2. Subdomains are color-coded and include N-terminal ubiquitin-like domain in dark blue, thumb subdomain in light blue, palm domain in red and fingers domain in orange. Image obtained by Osipiuk et al., 2021 [60], PDB entry: 7JIW.

Along with its protease activity, PL^pro^ can modulate the innate immune response by cleaving ubiquitin and interferon-stimulated gene 15 (ISG15), known regulators of host innate immune pathways. Amino acid sequences of PL^pro^ from SARS-CoV and SARS-CoV-2 are 83% identical. Interestingly, PL^pro^ from SARS-CoV and SARS-CoV-2 do not have the same specificity. The one from SARS-CoV acts more specifically on ubiquitinated substrates, and the one from SARS-CoV-2 preferentially acts on ISG15-conjugated substrates. Inhibition of this protease stops viral replication [59]. The structure of PL^pro^ from SARS-CoV 2 is presented in Figure 5.

This protein is slightly basic, and it contains a high amount of Cys residues. Cys111 is part of the catalytic triad that is formed from Cys111, His272, and Asp286. Additionally, there are four Cys residues involved in the coordination of Zn ion that is very important for the enzyme structure and six more Cys residues located through the enzyme. The enzyme has a structure similar to ubiquitin-specific proteases composed of “thumb–palm–fingers” catalytic domain and N-terminal ubiquitin-like domain. Zn ion is located in the fingers subdomain and is coordinated by Cys189, 192, 224, and 226, while the catalytic site of this enzyme is located at the interface of palm and thumb subdomains [60].

### 3.2. Antiviral Activity of Polyphenols

The increasing trend of scientific interest in polyphenol activities over the past two years has resulted in the investigation of several strategies pertaining to their antiviral effect against SARS-CoV-2. Vaccines for the prevention of COVID-19 have significantly lowered the burden of disease management. Still, pharmacological treatment options are incessantly being tested [61]. Despite concerted efforts invested in the development of new drugs, it is well-known that the time lapse between the drug discovery and its clinical approval might be long. Subsequently, repurposing of existing drugs might serve as a better alternative. To date, several screening efforts have been performed in an attempt to identify both drug repurposing targets and possible antiviral compounds among those already in use for other purposes [62,63]. A recently published drug repurposing study screened more than 1400 FDA-approved drugs and compounds using artificial intelligence-powered morphological analysis of several human cells lines during infection with SARS-CoV-2. They identified 17 candidate compounds including one dietary supplement, that have been shown to block or reduce SARS-CoV-2 infection in cells [64]. In this context regarding the potential anti-SARS-CoV-2 activity, polyphenols, common dietary supplements have also been proposed as one of the alternatives to combat against this global health crisis [27,65,66].

**Figure 6:**
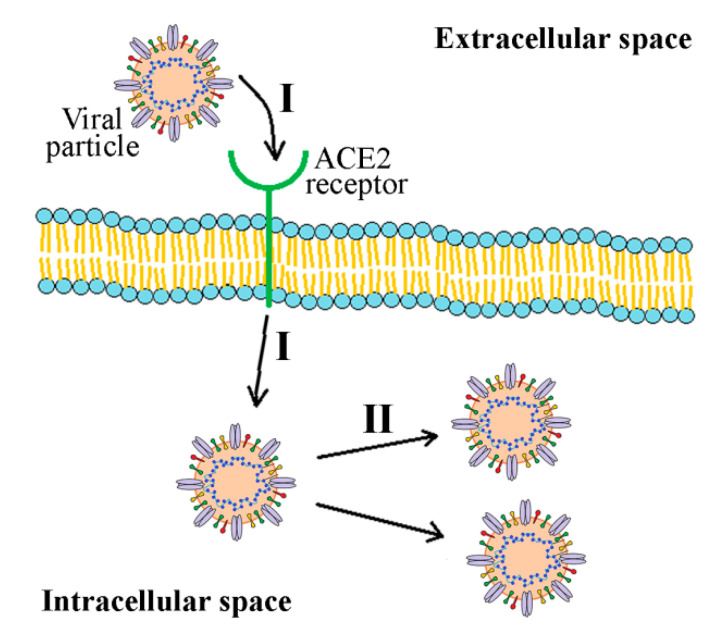
Entrance of SARS-CoV-2 virus in a host cell via interaction with ACE2 receptor (**I**) and its subsequent internal replication (**II**). Polyphenols are shown to inhibit both modes of action.

In addition to their well-known antioxidative, anti-inflammatory, and immunomodulatory properties tending to influence viral infection outcomes by upregulating the body’s immune system [67], polyphenols’ direct anti-SARS-CoV-2 activity has also been reinforced due to their demonstrated inhibitory action against replication in other coronaviruses, such as SARS-CoV and MERS-CoV [23]. Interactions of polyphenol with viral proteins and/or host cell receptors may interfere with the entry of the virus and its replication in the host cell (Figure 6). In this regard, the greatest focus has been put on the inhibition of viral proteases and the prevention of S protein-ACE2 interaction.

The numerous computational approaches carried throughout the emergence of the novel coronavirus speculated on the potential of different polyphenols to interact with SARS-CoV-2 viral proteins, thus, providing a platform for future in vitro and in vivo studies. Besides in silico analyses, the antiviral effect of polyphenols has also been demonstrated in vitro. Polyphenols may directly disrupt the life cycle of viruses by binding to their selected essential proteins. These interactions could either inhibit viral enzymes, such as 3CL^pro^ or disturb the binding of viral structural proteins, for example, S protein, to host cell proteins. Polyphenols have also shown to affect expression levels of different proteins, which could also aid to their antiviral effect [6,68]. Previously discovered protein targets for polyphenols include viral proteins, such as 3CL^pro^ and S protein, and some of the host cell proteins, including TMPRSS2, SIRT1, ACE2 receptors, and dipeptidyl Peptidase 4 (DPP4) [27]. Although, DPP4 is another host cell receptor for the SARS-CoV-2 virus, its binding affinity for S protein is weaker than that of the ACE2 receptor. The clinical data regarding the usage of DPP4 inhibitors and/or their potential adverse effect on patients with COVID-19 is scarce [69].

The following section will give an overview of in vitro data obtained so far on the effects of various polyphenols on main viral protein targets. Obtained in vitro IC_50_ values for different polyphenols against 3CL^pro^ and PL^pro^ are given in Table 2.

#### 3.2.1. CL^pro^/M^pro^ and PL^pro^ as Targets

Having no closely related homolog in humans, the main protease of SARS-CoV-2 (M^pro^/3CL^pro^) has been an important target for drug development due to its crucial role in polyprotein processing and virus maturation [57]. To date, numerous polyphenols from different natural sources with recognized antiviral activities have been screened in silico for their potential to inhibit SARS-CoV-2 M^pro^ activity.

Among several polyphenols investigated in the study by Ghosh et al., epigallocatechin-3-gallate (EGCG), epicatechin-gallate, and gallocatechin-3-gallate demonstrated good binding affinity toward M^pro^ while also being able to interact with one or both of its catalytic residues (His41 and Cys 145) by hydrogen bonding. As revealed by molecular dynamics (MD) simulations, formed complexes were highly stable and less prone to conformational fluctuations in comparison to the unligated enzyme [68]. In another study, Singh and coauthors identified three structurally similar polyphenolic compounds, namely mangiferin, glucogallin, and phlorizin, as potential M^pro^ and host protein TMPRSS2 protease inhibitors. Molecular docking showed good binding affinities, while the MD simulation study predicted that these compounds could significantly stabilize the binding cavity of the M^pro^ and TMPRSS2 of SARS-CoV-2, thus possibly preventing virus maturation and S protein priming [70]. A comprehensive in silico study by Vijaykumar and coauthors identified 30 compounds capable of interfering with activation/dimerization of the M^pro^ and 40 compounds acting at M^pro^ regulatory sites, thus possibly lowering enzyme efficiency [71].

While in silico approaches do not necessarily guarantee antiviral behavior, they encourage and pave the way for future in vitro and in vivo studies, particularly if performed with ligands containing proven antiviral activities towards homolog proteins of SARS-CoV-1 and other viruses. For instance, quercetin, a water-soluble flavonoid, is a well-established antiviral agent for dengue and influenza A viruses [72,73]. Quercetin and its naturally occurring and synthetic analogs have displayed inhibitory activity towards the M^pro^ of SARS-CoV. Quercetin-3-β-galactoside has also been identified as a protease inhibitor [74]. Among natural compounds tested in silico for their interactivity with SARS-CoV-2 M^pro^, RNA dependent RNA polymerase (rdrp) and S protein, quercetin showed strong interactions with M^pro^ and the receptor-binding domain of the S protein [71]. Evidently, in yet another study conducted by Abian and colleagues, quercetin inhibited the activity of M^pro^ from the SARS-CoV-2 virus. The inhibition constant, K_i,_ was calculated to be 7 µM, justifying quercetin as a potent enzyme inhibitor. The dissociation constant determined by isothermal calorimetry was 2.7 μM in the absence of NaCl and 10 μM in the presence of 150 mM NaCl [75]. Despite a high homology between the SARS-CoV and SARS-CoV-2 main proteases, IC_50_ concentrations for the same polyphenol could still differ, for instance, quercetin has an IC_50_ of 73 ± 4 µM for the main protease in SARS-CoV [76] and 21 µM for the main protease in SARS-CoV-2 [75]. Docking simulation scores were also different for these two proteases.

Along with inhibitory activity against M^pro^, quercetin also inhibited recombinant human ACE2 of SARS-CoV with IC_50_ values similar to those for the inhibition of viral M^pro^ protease. The inhibition was time-dependent wherein shorter incubation times resulted in higher inhibitory activity. This is not a desirable effect since inhibition of ACE2 is shown to impact negatively on viral recovery [77] and hence highlights the need for a comprehensive analysis of any polyphenol with potential antiviral activity. It is essential to investigate their exact mode of action and whether their anti-inflammatory abilities could surpass the pro-inflammatory effect due to inhibition of ACE2 activity [78].

Flavonoids herbacetin, rhoifolin, and pectolinarin were previously found to be effective inhibitors of M^pro^ of SARS-CoV. Using FRET-based assay, IC_50_ values of compounds were determined and were 33.17, 27.45, and 37.78 µM, respectively. They were predicted to bind at the active site of the main viral protease [79]. This study was continued with the screening of the flavonoid library for inhibitory actions against SARS-CoV-2 M^pro^ activity, and it was discovered that herbacetin, pectolinarin, and baicalin block the proteolytic activity of SARS-CoV M^pro^ [80].

Tannic acid was revealed to be another potential natural drug against SARS-CoV-2. This polyphenol acts as a dual inhibitor, inhibiting both M^pro^ and host cell protease TMPRSS2. By using surface plasmon resonance (SPR), tannic acid illustrated binding to M^pro^ with a dissociation constant of 1.1 μM and TMPRSS2 with a dissociation constant of 1.77 µM. The concentration of tannic acid required to inhibit 50% of the proteases activity, IC_50_, was 13.4 μM for M^pro^ and 2.31 μM for TMPRSS2. As assessed by luciferase reporter assay, tannic acid also proved to inhibit the entrance of SARS-CoV-2 pseudovirus in the human embryonic kidney cell line 293T which firmly expresses the recombinant human ACE2 receptor and in African green monkey kidney Vero E6 cells. Favorably, tannic acid showed little to no cytotoxicity towards tested cells [81].

Curcumin, being already explored in vitro as a potent inhibitor of SARS-CoV M^pro^ with an IC_50_ value of 20 µM [82], is considered a promising natural polyphenol for mitigating the severity of COVID-19 disease. Indeed, the concentrations of curcumin above 30 µg/mL reduced the activity of SARS-CoV 2 M^pro^ by more than 50%, while the higher concentration of 75 µg/mL produced residual activity of 28.1% [83]. Besides M^pro^, this molecule can also inhibit PL^pro^ from SARS-CoV, with an IC_50_ of 5.7 µM [82]. In a recent study, docking simulation showed that curcumin might covalently bind to Cys111 in the active site of PL^pro^ by Michael’s addition reaction, thus inhibiting this enzyme [84]. Curcumin-loaded nanocarriers aimed to increase its bioavailability are currently being explored as antiviral alternatives. They are proposed to enhance curcumin bioavailability and act as antiviral agents themselves, possibly displaying synergistic effects in combination with curcumin [85].

Eckol and dieckol, phlorotannins isolated from edible brown algae *Ecklonia cava*, inhibited M^pro^ from SARS-CoV. The cell-free, FRET-based assay showed that these two compounds had IC_50_ values of 8.8 ± 3.5 and 2.7 ± 0.6 µM towards M^pro^ from SARS-CoV, respectively. On the other hand, the cell-based inhibition assay, or the so-called *cis*-cleavage assay, showed that only dieckol effectively inhibited M^pro^ with an IC_50_ of 68.1 ± 2.2 µM. The explanation for this could be that this compound penetrates the cellular membrane more effectively and reaches M^pro^ better than eckol [86]. Based on these results, their potential as effective competitive inhibitors for SARS-CoV-2 M^pro^ has been suggested [66].

EGCG, the primary polyphenol of green tea, has been identified as a potential inhibitor of SARS-CoV-2 M^pro^ in the recent in silico molecular docking study [68], with its antiviral effect later being demonstrated in vitro as well. In support of the claims mentioned above about differences in the behavior of the same polyphenol against SARS-CoV and SARS-CoV-2 enzymes, the inhibition efficiency of EGCG towards M^pro^ from SARS-CoV was different than the one towards M^pro^ from SARS-CoV 2. In their study, Nguyen and colleagues found that epigallocatechin gallate and especially gallocatechin gallate were more potent inhibitors of M^pro^ from SARS-CoV [76] than the same protease from SARS-CoV-2 [87]. It should be noted that the authors used recombinant SARS-CoV M^pro^ expressed in *P. pastoris* and SARS-CoV-2 M^pro^ expressed in *E. coli*, a factor that could influence obtained results. In a study by Jang and coauthors, EGCG and theaflavin, the primary polyphenol in black tea, inhibited the activity of M^pro^ of SARS-CoV-2 with IC_50_ of 16.5 µM for EGCG and 14.9 µM for theaflavin. The authors used M^pro^ expressed in *E. coli* and FRET-based protease assay for enzyme activity. As EGCG is susceptible to oxidation, the inhibitory activity of its auto-oxidation products was also examined. Notably, after 12 h of auto-oxidation of EGCG, there was no significant impact on its inhibitory activity. However, the inhibitory activity of EGCG has decreased following 24 h oxidation, although some of the inhibitory effect was still retained. Considering that maximal physiologically obtainable concentrations of EGCG and theaflavin are significantly lower than their IC_50_ values, retention of some of the EGCG inhibitory activity even after 24 h might be beneficial for potential antiviral activity of EGCG. Moreover, the authors have also explored whether the combined use of EGCG and theaflavin has a synergistic effect on inhibition of M^pro^ activity but showed only an additive effect [88].

In a study investigating the inhibitory activity of 49 polyphenols towards SARS-CoV-2 M^pro^, besides already mentioned tannic acid, isoflavones puerarin and daidzein and flavonol myricetin were found to be potent inhibitors of this enzyme with IC_50_ values of 42, 56, and 43 µM, respectively. In addition, their mixtures with tannic acid proved to be effective as well. This may be a helpful finding since it reduces each polyphenol’s required amount for obtaining the desired effect [87]. In a study by Xiao et al., myricetin was also identified in vitro as a potent inhibitor of M^pro^ but with an order of magnitude lower IC_50_ value of 3.684 ± 0.076 μM. Molecular docking showed that myricetin could bind at the active site of M^pro^. Additionally, myricetin improved bleomycin-induced pulmonary inflammation, suggesting that it may be used both as an antiviral drug and for symptomatic treatment of COVID-19 [89]. Myricetin has also shown potent inhibitory activity towards SARS-CoV-1 nsP13 helicase, together with scutellarein. They were able to inhibit ATPase activity but not DNA unwinding activity of viral helicase. ATPase activity was determined by measuring the formation of inorganic phosphate in a colorimetric assay using molybdate [90]. Whether these two flavonoids could inhibit the activity of helicase from SARS-CoV-2 remains to be seen.

Different IC_50_ values for EGCG obtained by Nguyen et al. and Jang et al. and those for myricetin obtained by Nguyen et al. and Xiao et al. indicate that experimental results should be interpreted cautiously since differences in experimental setup could significantly influence obtained results.

Chalcone isolated from *Angelica keiskei*, named xanthoangelol E, was shown to be an effective inhibitor of both M^pro^ and PL^pro^ proteases of SARS-CoV with IC_50_ values of 11.4 µM and 1.2 µM, respectively. These values were obtained using recombinant *E. coli* enzymes and an inhibitory FRET assay. Chalcone competitively inhibited M^pro^, while PL^pro^ was inhibited in a non-competitive manner. The calculated IC_50_ concentrations were below CC_50_ (50% cytotoxic concentration), which for this polyphenol was determined to be 65.6 µM [91]. Isolated polyphenols from *Broussonetia papyrifera*, broussochalcone A, broussochalcone B, papyriflavonol A, 3′-(3-methylbut-2-enyl)-3′,4,7-trihydroxyflavane, 4-hydroxyisolonchocarpin, kazinol A, kazinol B, kazinol F, kazinol J, and broussoflavan A were tested as potential inhibitors of both M^pro^ and PL^pro^ proteases of SARS-CoV. In general, tested polyphenols were more potent inhibitors of PL^pro^, with papyriflavonol A being the best with a calculated IC_50_ of 3.7 µM for this enzyme. On the other hand, the best inhibitor of M^pro^ was 3′-(3-methylbut-2-enyl)-3′,4,7-trihydroxyflavane [92].

Ethanol extract from *Psoralea corylifolia* seeds was shown to have inhibitory activity towards SARS-CoV PL^pro^ with IC_50_ value of 15 µg/mL. Further analysis showed that this extract contained six phenolic compounds: bavachinin, neobavaisoflavone, isobavachalcone, 4′-*O*-methylbavachalcone, psoralidin, and corylifol A. All of these compounds were reversible, mixed type inhibitors for PL^pro^, with IC_50_ values of 38.4 ± 2.4, 18.3 ± 1.1, 7.3 ± 0.8, 10.1 ± 1.2, 4.2 ± 1.0, and 32.3 ± 3.2 µM, respectively [93].

To the best of our knowledge, currently, there are no in vitro data on the antiviral activity of polyphenols towards PL^pro^ of SARS-CoV-2. A noticed lack of in silico studies has been attributed to the paucity of known small molecule inhibitors of SARS-CoV-2 PL^pro^ as well as the lack of structural conformation of the SARS-CoV-2 PL^pro^ binding site when bound to conventional inhibitors [94]. A molecular dynamics simulation study by Huynh and coauthors suggested that chances for drug repurposing might be low in case of PL^pro^ [95]. Therefore, polyphenols with inhibitory activity against SARS-CoV PL^pro^ might not be as effective against SARS-CoV-2 PL^pro^.

**Table 2:** In vitro IC_50_ values for different polyphenols against 3CL^pro^ and PL^pro.^

Enzyme	Polyphenol	IC 50 (µM)		Reference
		SARS-CoV	SARS-CoV-2	
**3CL^pro^**	Quercetin	73 ± 4	21	[75,76]
	Gallocatechin gallate	47	N/A	[76]
	Herbacetin	33.17	53.9	[79,80]
	Rhoifolin	27.45	N/A	[79]
	Pectolinarin	37.78	51.64	[79,80]
	Baicalin	N/A	34.71	[80]
	Tannic acid	N/A	13.14	[81]
	Curcumin	20	N/A	[82]
	Eckol	8.8 ± 3.5	N/A	[86]
	Dieckol	2.7 ± 0.6	N/A	[86]
	Epigallocatechin gallate	73	16.5, 171 ± 5	[76,87,88]
	Theaflavin	N/A	14.9	[88]
	Puerarin	N/A	42	[87]
	Daidzein	N/A	56	[87]
	Myricetin	N/A	43, 3.68 ± 0.08	[87,89]
	Xanthoangelol E	11.4	N/A	[91]
**PL^pro^**	Curcumin	5.7	N/A	[82]
	Xanthoangelol E	1.2	N/A	[91]
	Papyriflavonol A	3.7	N/A	[92]
	Bavachinin	38.4 ± 2.4	N/A	[93]
	Neobavaisoflavone	18.3 ± 1.1	N/A	[93]
	Isobavachalcone	7.3 ± 0.8	N/A	[93]
	4′-*O*-methylbavachalcone	10.1 ± 1.2	N/A	[93]
	Psoralidin	4.2 ± 1	N/A	[93]
	Corylifol A	32.3 ± 3.2	N/A	[93]

#### 3.2.2. S Protein and S-ACE2 as a Target

An in-depth in vitro study by Goc and co-workers examined the effect of 56 polyphenols on the binding of viral S protein for ACE2 receptor and its fusion for the cell membrane. They showed that brazilin, theaflavin-3,3′-digallate, and curcumin had the strongest binding to the RBD domain of S protein. Both cell-based and cell-free experiments confirmed this finding. These compounds were also able to reduce the fusion of cells expressing the S protein to the human ACE2 overdressing cellular monolayer. Theaflavin-3,3′-digallate and curcumin not only inhibit the ACE2 activity in both cell-free and cell-based assays but also bind the human ACE2 receptor (hACE2) with moderate affinity. These effects were not shown for brazilin. None of these three molecules affected the expression of hACE2 in human alveolar epithelial A549 cells. Inhibition of host proteases, TMPRSS2 and cathepsin L, was also observed, which is essential for cleavage of S protein and subsequent viral fusion to host cell membrane [96].

Pomegranate peel extract in ethanol/water (70/30 *v*/*v*), rich in polyphenols, was investigated for its potential to inhibit SARS-CoV-2 activity by acting on viral S protein and ACE2 receptor. The abundant polyphenols in the obtained extract were ellagitannins punicalagin, punicalin, granatin B, causarinin, galloyl-HHDP-hexoside, pedunculagin I, and pedunculagin II, punicalagin being the most abundant. Based on in vitro results, pomegranate peel extract could bind to both S protein, presumably by interacting dominantly with the RBD domain, and to ACE2 receptor, but with 10-fold lower affinity. Interaction between S protein and ACE2 was inhibited on a cellular level. By using S protein-carrying Lentivirus, peel extract inhibited its entry into human kidney-2 cells. The extract was also able to downregulate the expression of both ACE2 and TMPRSS2 [97]. Although both proteins are involved in viral entry into the host cell, they also have an essential role in metabolic processes, so their downregulation might prove to be the pitfall. It was noticed that SARS-CoV infections and the S protein downregulate ACE2 expression, contributing to the severity of lung pathology [77].

Anthraquinone emodin, produced in higher levels in *Rheum* and *Polygonum* genus, effectively blocked the interaction between SARS-CoV S protein and ACE2 receptor. The compound inhibited the binding of S protein to ACE2 coupled to the microtiter plate in a competition ELISA assay, with an IC_50_ value of 200 µM. It also blocked the binding of S protein to Vero E6 cells expressing ACE2. Infectivity of S protein-pseudotyped retrovirus to ACE2-expressing Vero E6 cells was also reduced. Inhibition of viral infectivity for 50 µM of emodin was 94.12 ± 5.90% [98]. Emodin has emerged as a potential anti-SARS-CoV-2 compound in a comprehensive drug repurposing screening [62]. A recent molecular docking study dealing with the mechanistic investigation of the interaction between SARS-CoV S protein and emodin proposed that emodin could bind at the interface of two interacting proteins, thus destabilizing their interaction. A similar surface pocket was found at the contact surface of the SARS-CoV-2 S protein–ACE2 complex, providing evidence of the possibly similar activity of emodin against SARS-CoV-2 [99].

Among polyphenols investigated for their anti-SARS-CoV-2 activity, a great interest has been put on resveratrol. Its antioxidant and anti-inflammatory activities, particularly concerning cardiovascular health, have been reviewed extensively [100,101]. Along with its beneficial cardiovascular effects that may potentially reduce COVID-19 illness severity in patients having cardiovascular complications [102], resveratrol was also shown to directly interfere with the main pathways involved in SARS-CoV-2 pathogenesis including expression of ACE2 and regulation of the renin-angiotensin system [103]. In addition, the antiviral activity of resveratrol has been well documented against RNA viruses such as influenza virus, rhinovirus, Zika virus, and MERS-CoV [104,105,106,107]. Antiviral activity of resveratrol against MERS-CoV is associated with the promotion of cell survival and the reduction of MERS-CoV-induced apoptosis [107]. Resveratrol upregulates the expression of ACE2 in vascular smooth muscle cells stimulated by angiotensin II [108], which seems to be beneficial for mitigation of COVID-19 disease, as ACE2 has organ-protective effects in COVID-19 patients with poor prognosis [109]. A molecular docking study indicates that resveratrol binds to the S protein-ACE2 complex [110], which could be its inhibitory mode of action. The direct anti-SARS-CoV-2 effect of resveratrol has also been reported in several studies. Resveratrol was shown to inhibit the replication of SARS-CoV-2 in cultured Vero cells (ATCC, CCL-81). By utilizing both qRT-PCR and immunofluorescence assay, an IC_50_ of 4.48 μM was determined. Interestingly, resveratrol exerted higher inhibitory activity after the previous infection of cells with the virus. It was suggested that resveratrol could block viral entry into the cells [111]. In line with this, upon treatment with resveratrol, a reduced viral titer of SARS-CoV-2-infected Vero cells was reported by Pasquereau et al. with an IC_50_ value of 10.66 µM without significantly affecting cell viability [112]. Ter Ellen et al. showed that both resveratrol and its metabolically more stable dimethylated structural analog, pterostilbene, inhibited the replication of SARS-CoV-2 in air–liquid interface cultured human primary bronchial epithelial cells up to 48 h post-infection. Pterostilbene even inhibited SARS-CoV-2 replication more effectively than resveratrol [113]. Pterostilbene’s improved biological activities compared to resveratrol have been ascribed to its dimethyl ether structure, which increases its lipophilicity, thus limiting its glucuronidation and sulfation and improving solubility, absorption, and bioavailability [114].

EGCG, previously mentioned as a potential M^pro^ inhibitor, has also been shown to interact with viral S protein subunits, including S2 and RBD domains of both original and UK strain mutant virus, thus interrupting interaction with ACE2. EGCG binds to the RBD domain of the UK strain mutant slightly more effectively [115].

In vitro cytotoxic test using Vero E6 cells showed that phenols ferruginol and 7β-hydroxydeoxycryptojaponol were able to abolish the cytopathogenic effect of SARS-CoV 1 at concentrations of 10 and 20 µM. These phenols inhibited viral replication in the same cells, with IC_50_ values being 1.39 and 1.15 µM, respectively. Obtained IC_50_ values for the inhibition of viral replication were much lower than their CC_50_ values, 80.4 µM for ferruginol and 127 µM for 7β-hydroxydeoxycryptojaponol [82]. It remains to be seen if ferruginol is also effective against SARS-CoV-2.

## 4. Clinical Studies on the Role of Polyphenols in the Treatment of COVID-19 Patients

In a race to prevent the spread of COVID-19 and to stop the global pandemic, it is no surprise that many researchers and MDs have turned their attention to natural compounds, which are safe for humans and have a history of therapeutic usage. Therefore, other than the drugs being tested in clinical trials such as hydroxychloroquine, favipiravir, remdesivir, and lopinavir/ritonavir [116], well-characterized polyphenolic compounds with previously established anti-viral and anti-inflammatory properties are also among the candidates evaluated for COVID-19 treatment or prophylaxis.

### 4.1. Green Tea Polyphenols as an Addition to Standard of Care Therapy for COVID-19

The lack of therapies for COVID-19 patients with mild and moderate symptoms compelled a group of researchers from the Istituto Nazionale Biostrutture e Biosistemi, Italy, to use standardized polyphenolic green tea extract during the lockdown in Italy in Autumn 2020 [117] to treat ten symptomatic COVID-19 patients with positive nasopharyngeal swab tests for the SARS-CoV-2 virus. The group already had experience in green tea extract application and dosage in animal [118] and clinical studies [119] related to vascular and cancer disease. The green tea extract they chose for the COVID-19 study was Theaphenon E (ThE), the improved or new version of the Polyphenon E (PoE) manufactured by the Mitsui Norin Co., Ltd., Shizuoka 426-0133 Japan, which is composed mainly of green tea catechins (85–95%) out of which EGCG accounts for 65–70% of the total catechins [120]. PoE has been used in 35 clinical trials in the U.S. (Medicine) and was approved by the U.S. FDA in 2006 as a tropic drug for warts caused by the papilloma virus [121]. The therapy lasted for 15 days and consisted of two sessions of inhalation (0.3% ThE in 5 mL sterile PBS pH 5.8 per nebulization session, 19 mg/day of EGCG) plus three capsules per day (300 mg ThE/capsule or 595 mg/day of EGCG) accounting for a total of 840 mg of catechins or 595 mg of EGCG per day, which was below the safety threshold limit suggested by the European Food Safety Agency of 800 mg of EGCG per day [122].

Before testing the efficacy of ThE on COVID-19 patients, Bettuzzi et al. [117] tested the proposed therapy in two healthy volunteers with no side effects. All COVID-19 patients started the ThE therapy at a median of 5 days within a range of 3–6 days. Blood samples were taken by their family doctor at the study enrollment date (day 0) and later when the symptoms were gone at a median of 9 days (range of 7–15 days). Seven patients were negative in a SARS-CoV-2 swab test at a median of 9 days (range of 6–13 days), 1 out of the 3 still swab-positive patients had a very low viral load. At the end of the therapy the inflammation markers monitored in the study had significantly decreased: α-1 anti-trypsin, C-reactive protein and eosinophils in all patients, IL-6 and erythrocyte sedimentation rate in 7 out of 10 patients. The authors compared the efficacy of the proposed treatment with the study data reported by Manusco et al. [123], who followed 4480 patients with a positive SARS-CoV-2 swab test in Italy from 26 of February to 22 of March 2020. Manusco et al. [123] reported that at day 10, 0.7% of the patients had a first negative swab test, with the percentage increasing to 19.0% on day 20. It seems that green tea polyphenol treatment might have helped clear the virus from the patients, as 20% of patients from the Bettuzzi et al. study [117] had the first negative swab at 10 days, increasing to 70% at day 20 from the first swab. As the authors declared, the effect of green tea polyphenols was noticeable, as the probability of this happening by chance was *p* < 0.0023 at day 10 or *p* < 0.005 at day 20 (one-tailed Fisher-Yates test performed by the authors of the study), but the mechanism was unknown. As thoroughly discussed above in our paper, anti-inflammatory and anti-viral effects of green tea polyphenols are known, and there is a growing body of computational and in vitro data suggesting the formation of specific interactions with SARS-CoV-2 proteins, it seems to us that the key to the success of this study was the way the polyphenols were delivered: at high dose, orally, targeting the SARS-CoV-2 infection in the gastrointestinal tract, and at a lower dose, pulmonary, targeting the virus and the immune response in lungs directly. The administration of green tea polyphenols through inhalation is not new. It was safely administered to 48 elderly and hospitalized patients with methicillin-resistant Staphylococcus aureus infection in a study by Yamada et al. [124]. To further clarify if green tea polyphenols can aid in speeding up the recovery and preventing the death of COVID-19 patients, a clinical trial with a higher number of enrolled subjects is required.

### 4.2. Curcumin with Piperine as an Addition to the Standard of Care Therapy for COVID-19

As discussed previously in the paper, curcumin from turmeric might be beneficial in treating COVID-19. There are preclinical and clinical studies showing its positive effect on the treatment of many disorders, including but not limited to gastrointestinal, inflammatory, respiratory, and cardiovascular diseases [125,126]. A group from India performed a double-blind, randomized, controlled trial (CTRI/2020/05/025482) in a COVID-19 dedicated hospital in Maharashtra, India, from July to September 2020, publishing the study results in May 2021 [127].

The group recruited adult symptomatic patients with a positive antigen test, further randomly divided into a control and study group, each accounting for 70 patients. To equalize the distribution of patients to the natural occurrence of mild, moderate, and severe symptoms, according to their pilot study, each group had 30 patients with mild, 25 with moderate, and 15 with severe symptoms. Patients with mild symptoms had oxygen saturation SpO2 > 94%, those with moderate symptoms had SpO2 between 90 and 94 and pneumonitis, and severely ill were those with SpO2 < 90%. In addition to the standard of care for COVID-19 in India, the control group received a probiotic capsule, and the study group received a dietary supplement of curcumin in the form of Curcumin C3 Complex^®^ (SamiDirect, Bangalore, India) approved for human use by the U.S. FDA, together with an absorption adjuvant piperin (Bioperine^®^, SamiDirect, Bangalore, India). The therapy lasted for 14 days, during which 525 mg of curcumin and 2.5 mg of piperine were administered orally 2 times a day. Serology testing for complete blood count, C-reactive protein (CRP), and D-dimer was performed on admission and later if required.

Overall, the authors show that patients from the study group recovered earlier from COVID-19 symptoms than patients in the control group, with less deterioration of their condition over their period of the hospital stay and better oxygen saturation on room air with less patients needing oxygen therapy or mechanical ventilation. In the patients with mild symptoms, curcumin treatment significantly reduced the number of days the patients had SpO2 less than 94%, the number of patients requiring oxygen treatment, and the number of patients with a neutrophil/lymphocyte ratio of less than 3.5. CRP and D-dimer were maintained in this subgroup at the admission level, with no significant rise in each of them. Apart from significantly maintaining the oxygen saturation levels above 94% in patients with moderate symptoms, curcumin also significantly reduced the number of patients who required the use of COVID-19 awake repositioning/proning protocol, the number of patients requiring low molecular weight heparin, and the number of patients requiring remdesivir. D-dimer was maintained at the admission level, and there were significantly less patients with increased CRP levels. The numbers were not as pronounced in the severely ill patients, suggesting that the treatment might not work in this study subset.

Clinical data from this study are provided in detail as it was a study performed in a hospital. However, there are some data that could help us in understanding the mechanism of action that are missing, such as IL-6 levels, prothrombin time, and swab tests, but considering that the authors had limited resources, that is understandable. The most pronounced results from this study are the effect of treatment on oxygen saturation and D-dimer levels in patients with mild and moderate COVID-19 symptoms. Curcumin is known for its antithrombotic effects, and as the authors suggest, it might be considered for use with other blood thinners used in COVID-19 treatment protocols. When comparing this clinical study to the one described above, it seems that curcumin is less potent than green tea catechins against COVID-19, but that needs to be further elucidated using the same or similar study strategy and population size.

As the data from the in vitro studies are accumulating, and different animal models are being developed [128], we should be seeing more reports on the in vivo effects of polyphenolic compounds on SARS-CoV-2 infection and more clinical trials where they are used as an adjuvant therapy. So far, we have found only one publication in the form of a letter where authors tested Pudilan Xiaoyan Oral Liquid, a traditional Chinese medicine containing four herbs (Indigowoad Root (*Isatis indigotica*), Bunge Corydalis (*Corydalis bungeana*), Mongolian Dandelion (*Taraxacum mongolicum*), Scutellaria Amoena (*Scutellaria baicalensis*)) and more than 180 ingredients, including polyphenols in a mouse model. The herbal mixture exhibited potent anti-SARS-CoV-2 activity in infected hACE2 mice [129].

## 5. General Protective/Preventive Effects of Polyphenols in Diseases Related to COVID-19

**Figure 7:**
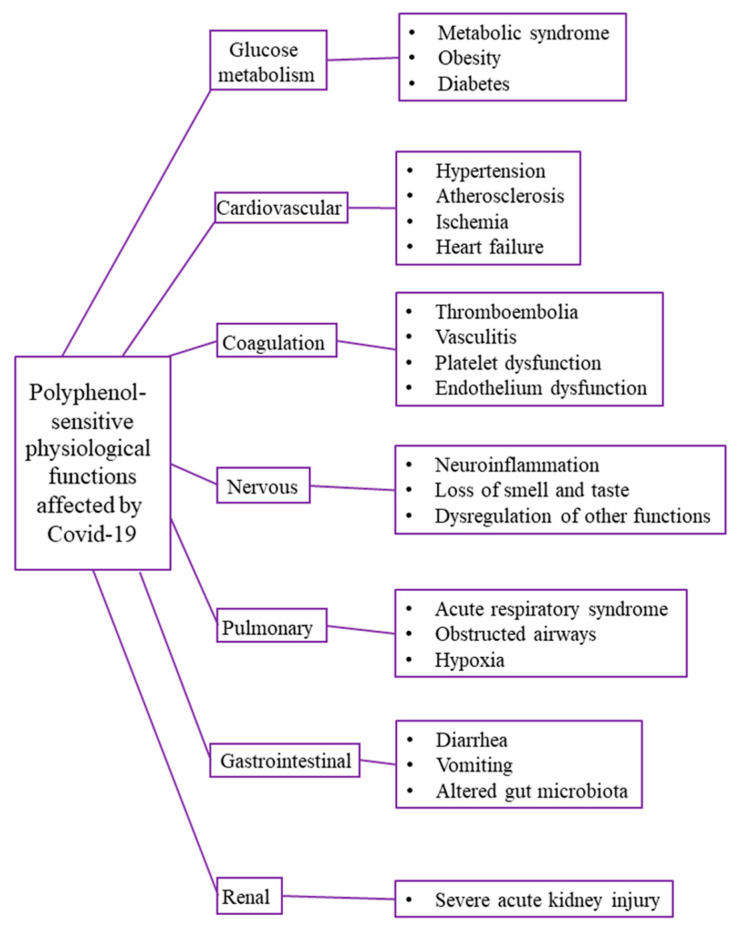
Physiological functions affected by COVID-19 and related diseases which the consumption of polyphenols can ameliorate.

COVID-19 induces numerous pathophysiological manifestations affecting respiratory, immune, hematological, renal, nervous, and gastrointestinal (GI) systems [27,130]. Pre-existing chronic diseases, specifically cardiometabolic impairments and obesity, represent risk factors linked to poorer prognosis in COVID-19 patients. Complications accompanying COVID-19 can lead to organ dysfunction, metabolic acidosis, sepsis, blood coagulation dysfunction, cardiac arrest, and death [25]. Polyphenols and polyphenol-rich plants have been widely tested and used for the general treatment of COVID-19 [23,117,131,132]. An update on completed or recruiting clinical trials based on plants, their isolated products, and functional foods against COVID-19 is summarized in an article by Alam et al. (2021) [133]. The protective effects of polyphenols against acute lung injury are highlighted in a review paper by He et al. (2021) [134], while a review of clinical trials data by Santos et al. (2021) [29] reinforces the potency of polyphenols in the treatment of COVID-19. Physiological functions affected by COVID-19 and related diseases that the consumption of polyphenols can ameliorate are shown in Figure 7.

Polyphenols, strong antioxidants, can neutralize the harmful reactivity of undesired or overproduced reactive oxygen and nitrogen species (ROS and RNS) that arise during metabolic processes in the organism. Under physiological conditions, approximately 2% of the oxygen consumed by aerobic cells is directed towards the formation of ROS/RNS [26]. Polyphenols also influence several immunomodulatory pathways, thus regulating immunity. Numerous epidemiological studies have documented positive effects of polyphenols against the development of chronic diseases such as metabolic syndrome, diabetes, hypertension, cardiovascular diseases (CVD), infections, asthma, cancer, neurodegenerative diseases, and coagulopathies. By alleviating chronic disturbances and impairments, polyphenols also act as antiaging agents. Many of them have been found to exterminate senescent cells as well, therefore adjusting the inflammaging and immunosenescence [29]. Polyphenols have been confirmed as antimicrobial agents, affecting various levels of defense mechanisms against pathogen invasion. In addition, polyphenols exert prebiotic, pro-apoptotic, anti-proliferative, hormonal control [23], and anti-arthritic properties [135].

Thousands of polyphenolic compounds have been identified in various plant species [136]. Usually, a mixture of polyphenolic compounds is found in a plant, and a specific compound may be located only in one plant compartment (root, stem, leaf, flower, seed) or in a number of them. Polyphenol supplements are widely consumed as nutraceuticals. However, they are traditionally absorbed via polyphenol-rich plants and their derivatives in the form of juice, tea, and infusion. Polyphenols are constituents of functional foods. The main task of functional foods is to strengthen physiological functions. Since polyphenols participate in redox reactions, one should bear in mind that their antioxidant capacity is reduced upon improper or prolonged storage.

The bioavailability of each polyphenol differs, and there is no relation between its quantity in a plant/food and bioavailability, as a complex process of deconjugation, hydrolysis, gastrointestinal absorption, and further transformation occurs in the body [137]. Polyphenols undoubtedly exert health-improving effects, but little is known about their metabolic products or the exact metabolic mechanism of their action. Likewise, biological properties differ greatly from one polyphenol to another [136]. Common to all of them is the ability of the phenolic group to accept an electron, forming a relatively stable phenoxyl radical, thus, interfering with cellular oxidation reactions. Polyphenol metabolites in blood bind to proteins, particularly albumin. It is still unresolved whether the albumin-attached polyphenols can also perform physiological actions or in their free form exclusively. The half-life of polyphenols and their metabolites in the blood is short, as they are efficiently removed. For example, the half-life of resveratrol is approximately 10 h [138]. To enhance their biological half-life, increase oral absorption, or render them efficient locally, polyphenols are conjugated with other molecules, such as oligosaccharides [139], packed in granules with lipids such as sunflower phospholipids [137], nanoencapsulated [140], or prepared in a nasal spray form [141].

### 5.1. Preventive Effects of Polyphenol against Metabolic Syndrome and Diabetes

Metabolic syndrome and diabetes are associated with impaired glucose metabolism. The most common consequences are glucose insensitivity, impaired insulin secretion, obesity, various metabolic disturbances, inflammation, CVD, etc. Prolonged exposure to high glucose concentration leads to structural changes of physiological molecules (i.e., non-enzymatic glycosylation or glycation), formation of advanced-glycosylation end-products (AGEs), and modification of cellular structures (e.g., membranes); afterward, it might advance to irreversible retinopathy, nephropathy, and neuropathy. Diabetic ketoacidosis, hyperosmolar hyperglycemic condition, and acute metabolic complications were recorded in patients with diabetes and COVID-19 [27]. According to the study by Tessier et al. (2021) [130], acute hyperglycemia might enhance the entry of SARS-CoV-2 into additional host cells via upregulation of the expression of angiotensin-converting enzyme (ACE2) in other tissues. However, reduced expression of ACE2 was previously reported by Reich et al. (2008) [142] in patients with diabetes type 2 and kidney dysfunction. Additional clinical studies are essential to resolve the inconsistency in findings.

Polyphenols act by inhibiting intestinal glycosidases and glucose transporters, leading to reduced glucose absorption in the gut. Catechin, epicatechin, epigallocatechin, epicatechin gallate, soybean isoflavones, tannic acid, chlorogenic acid, and saponins decrease intestinal transport of glucose [143]. Additionally, polyphenols such as resveratrol exert anti-diabetic effects by modulating the activity of sirtuins (SIRTs), NAD+ deacetylase, which is responsible for the entire glucose homeostasis [144], and by improving insulin sensitivity, possibly by affecting potassium channels in pancreatic beta-cells [145]. Ferulic acid ameliorates glucose response to insulin [146], while quercetin can suppress lipid peroxidation in diabetics [147]. Consumption of coffee and its most abundant polyphenol, chlorogenic acid, stimulates phosphorylation of the insulin receptor and downstream signaling pathways as well as major glucose transporter 4 gene, GLUT4, translocation, thus increasing intracellular glucose utilization [148].

Obesity is recognized as a risk factor in COVID-19 patients. At least 25% of patients who died in the United States following COVID-19 were obese [27]. Obese patients have a higher risk of developing severe pneumonia and often require mechanical ventilation as their respiratory mechanics is impaired [130]. Different cells in the adipose tissue secrete adipokines, cytokines, and chemokines which contribute to the overall inflammatory reaction. Polyphenols induce a reduction in body weight and adipose tissue inflammation [27].

### 5.2. Preventive Effects of Polyphenol against Cardiovascular Diseases

Hypertension and atherosclerosis are the most common events that lead to CVD. Atherosclerosis develops in arterial regions, which are affected by lesions. Atherosclerotic plaque consists of lipids, calcium, fibrin, and other blood components over time, narrowing the vessel and reducing the blood flow. The consequence is reduced oxygen and nutrient supply to the affected organ, the most serious being when developed in the heart and brain. One of the risk factors for atherosclerosis is the oxidation of low-density lipoproteins (LDL), which easily adheres and forms plaques. Polyphenols efficiently inhibit this process; in addition, they also contribute to preventing or ameliorating CVD by reducing inflammatory effects, improving endothelial function, exerting anti-coagulation and anti-platelet action, and increasing high-density lipoproteins.

Quercetin and resveratrol are among the most potent polyphenols in combat against CVD. Quercetin was shown to inhibit the expression of matrix metalloproteinase 1 (MMP1) and disrupt the atherosclerotic plaque [149]. Resveratrol inhibits LDL oxidation and the activity of cyclooxygenase 1 (COX1), which is responsible for tromboxane A2 synthesis, a molecule that acts as a vasoconstrictor and an activator of platelet aggregation [150]. Resveratrol is responsible for the “French Paradox” phenomenon: high-fat diet and smoking and yet low incidence of CVD among the French.

### 5.3. Preventive Effects of Polyphenol against Coagulopathies

COVID-19 is associated with a high rate of thromboembolic complications [151,152,153]. The venous coagulopathy incidence was found to be significantly elevated in intensive care unit (ICU) patients (around 45%) than in non-ICU patients (approximately 23%) and much higher than in critically ill patients with other viral respiratory infections. After their demise, autopsy studies of COVID-19 patients revealed pulmonary macroemboli, severe endothelial injury, microemboli, and increased angiogenesis [153]. The strong procoagulant stage is accompanied by an increased concentration of D-dimer (fibrinogen degradation product) and enhanced platelet activation. Mechanisms underlying COVID-19 coagulopathy are not yet fully understood, but the direct effects of the virus on pneumocytes and the endothelium, local and systemic inflammation, and disturbances in the hemostasis plus circulatory stasis from immobility seem to contribute to this complex interplay. The advanced stage of COVID-19 accompanied by thrombogenicity may lead to reduced platelet count and deficiency of coagulation factors, thus increasing the risk of bleeding. Numerous clinical trials investigate anticoagulant treatments [153], but definitive recommendations are still awaited.

Polyphenols have shown an inhibitory effect on platelet activation pathways, such as P2Y-ADP, glycoprotein VI-collagen, protease-activated receptor 1-thrombin, and COX1-thromboxane [154]. The chlorogenic acid from cherries, apples, and plums; polyphenols from pigmented rice (anthocyanins); and propolis-derived polyphenols have been found to retain the antiplatelet effect [155]. Quercetin, cyanidin, silybin, cyanin, catechin, and epicatechin inhibit thrombin amidolytic activity, among which the first three can also affect its proteolytic activity [156,157]. Honokiol, a tree lignan, was identified as the most potent polyphenol with anti-thrombin potential that can influence the activation of the SIRT family of proteins [158,159].

### 5.4. Preventive Effects of Polyphenol against Neural Diseases

Oxidative stress is an underlying cause of many neurological and neurodegenerative diseases. Neuro trauma can activate glial cells, neurons, and neuroimmune cells in the brain, which induces the release of neuroinflammatory mediators, leading to neuronal death and cognitive decline. Neuroinflammation of the brain can dysregulate respiratory, cardiac, and renal functions [27]. There is still a lack of effective therapies to treat neurodegenerative disorders. COVID-19 patients have commonly reported temporary sensory impairment (smell and taste). Luteolin can suppress systemic neuroinflammation and improve cognitive functions, and it was shown to be efficient in COVID-19 patients [160]. It can act through several mechanisms, the most important being inhibition of COVID-19-associated cytokine release from mast cells, an event involved in cytokine storm in severe cases of SARS-CoV-2 infection.

### 5.5. Preventive Effects of Polyphenol against Respiratory Diseases

The respiratory system is heavily affected by COVID-19. Here, it is worth mentioning that respiratory diseases are often accompanied by narrowed or obstructed airways, wherein increased consumption of apple and soybean polyphenols has been speculated to improve lung function [161]. Patients with COVID-19 are vulnerable to reactivation of the Herpes simplex 1 virus (HSV) infection causing secondary respiratory infection [162], and polyphenols have been reported to exert anti-HSV activity. Polyphenols are now under consideration as promising therapeutic agents against HSV viremia [163].

### 5.6. Preventive Effects of Polyphenol against Dysbalance in Gut Microbiota

The inflammation in the respiratory system may cause changes in gut microbiota [27]. The lung–gut axis appears to be crucial in respiratory diseases, especially during viral infections, although the mechanism of interactions remains unclear. COVID-19 is also associated with gastrointestinal disturbances such as diarrhea, nausea, and vomiting [164]. Application of probiotics and prebiotics, including polyphenols [165], might strengthen the immune system and minimize the virus-associated respiratory tract damage [27]. Non-digestible food ingredients that selectively promote the growth of functional bacteria have beneficial effects on the intestinal lining. Polyphenol transformation by gut microbiota results in metabolites that can both reduce pathogens and enhance favorable bacterial strains. Intracolonic microorganisms are essential for polyphenol metabolism to produce beneficial bioactive metabolites.

### 5.7. Polyphenols, Mediterranean Diet, and COVID-19

A typical lifestyle during the COVID-19 pandemic was lockdown, unhealthy diet, physical inactivity, and anxiety. This newly embraced lifestyle contributed to increased risk factors for numerous comorbidities. A Mediterranean diet consists predominantly of fruits, vegetables, cereals, and fish, and it is associated with nutraceuticals such as polyphenols, omega polyunsaturated fatty acids, vitamins, and minerals. A negative correlation was observed between adherence to the Mediterranean diet and the number of COVID-19-related deaths in 23 countries out of 25; Italy and Spain were profiled as outliers in the study since they had unproportionally high mortality rates despite the high Mediterranean adequacy index [166]. The governmental response, differences in health care systems, and socio-cultural factors have contributed to the high death rate in these populations regardless of their nutritional habits. This negative association was discovered when the data were adjusted to link well-being factors (such as income, education, housing, environment, and life satisfaction) and physical inactivity. Greene and coauthors stressed that despite their listed limitations regarding their study and methodology, their findings are still significant enough to venture further if the Mediterranean diet and other dietary approaches could reduce the risk for COVID-19 comorbidities.

## 6. Polyphenol Based Phytomedicines

In addition to their intrinsic biological activity, naturally occurring polyphenols provide a solid base for the design of semisynthetic analogs with potentially improved bioactivity. Knowledge of determinants influencing binding modes of polyphenols to protein targets is important from the aspect of designing functionally improved inhibitors. Molecular modeling-assisted design of eight new derivatives of quercetin-3-β-galactoside containing chemical modifications of its key binding determinants has previously helped identify the structure-activity relationship of this type of inhibitor against SARS-CoV [74]. A similar approach could be explored in the case of SARS-CoV-2 proteins. For instance, monocarbohydrate moiety in baicalin significantly influenced its binding mode to 3CL^pro^ of SARS-CoV-2 in in silico study [80]. In regard to this, molecular docking studies have shown the potential of two products, Linebacker and Equivir, to interfere with viral spike protein, helicase, and protease sites on ACE2 receptor, thus potentially blocking SARS-CoV-2 replication and infection. Linebacker, a synthetic, flavonoid-based compound, showed the ability to bind to ACE2 receptor. Molecular docking showed that the binding energy of Linebacker for ACE2 was higher compared to chloroquine which is currently being investigated in clinical trials as a possible prophylactic and therapeutic in the treatment of COVID-19. Phytomedicines based on mixtures of several naturally occurring polyphenols are also being tested. For example, Equivir, a mixture of hesperetin, myricetin, and piperine, has antiviral activity. Equivir acts by inhibiting intracellular adhesion molecule 1, helicase, ATPase, neuraminidase, and polymerase, which reduces and prevents viral entry and replication. All three polyphenolic components of Equivir are registered as Generally Recognized as Safe (GRAS) by FDA [167].

## 7. Conclusions and Future Perspectives

Literature data about the protective role of plant phenolic compounds as molecules against SARS-CoV-2 viral infection is on the rise, especially during 2021. Here, we reviewed the most recent advances related to molecular mechanisms of possible action of phenolic compounds in COVID-19 protection and prevention including the current views on phenolic compounds intervention at least as an adjunct therapeutic support. This review demonstrates that phenolic compounds may have several mechanisms of action against SARS-CoV-2. In addition to their antioxidant activities, multiple cellular and molecular pathways have been shown to be involved in their mechanism of action against different chronic disorders. However, there is still a lack of studies on the effects and mechanisms of in vivo-formed metabolites of phenolic compounds against SARS-CoV-2, although these metabolites are increasingly recognized as the main acting components in situ.

Further clinical studies, primarily in vivo, are still crucial to assess the therapeutic and pharmacological potential of phenolic compounds not just in the course but also post COVID-19 infection. Moreover, specifically designed clinical studies are necessary to understand individual variability of phenolic compounds action in COVID-19 infection, particularly considering their bioavailability. The epidemiological studies demonstrating a correlation between daily consumption of phenolic compounds and prevention of SARS-CoV-2 infection and development of complications would be valuable for nutritional recommendations and their application as nutraceuticals to combat COVID-19. Expansion of functional foods and pharmaceutical preparations based on phenolic compounds in the last decade should be exploited to raise population awareness of the multifaceted beneficial properties of phenolic compounds against COVID-19 and the significance of phenolic compound-enriched/or supplemented nutrition. The low risk of phenolic compounds interacting with concomitant pharmacological treatments and side effects make these compounds safe and thus suitable for their inclusion in clinical practice via the oral and respiratory routes by application of aerosol delivery systems.

Phenolic compounds-rich plant extracts have been in use since ancient medicine [168], and they are promising aids in SARS-CoV-2 prevention and treatment, particularly due to the synergistic effects of their components [169]; however, the standardization of extracts is necessary as plant extracts consist of a series of different compounds occurring in varying concentrations. In addition, further identification of individual phenolic compounds and determination of their particular mechanisms of action and their synergistic, additive, or complementary effects in COVID-19 prevention would be of great interest, although it is challenging.

This review shows that, with their multiple-target mechanism of action, phenolic compounds are promising adjuvants for prevention, treatment, and recovery strategies in the fight against COVID-19 multisystem pathogens.

## Figures and Tables

**Table 1:** Clinical trials investigating the effects of polyphenols in prophylaxis and the treatment of COVID-19.

Types of Polyphenol Combinations Used in Clinical Trials	ClinicalTrials.gov
Pure polyphenols	NCT04377789 NCT04578158 NCT04536090 NCT04446065 NCT04861298 NCT04851821 NCT04799743
Pure polyphenols in combination with vitamins/minerals and/or other natural bioactive compounds	NCT04468139 NCT04542993 NCT04507867 NCT04844658 NCT05008003 NCT04666753
Pure polyphenols in combination with drugs	NCT04622865 NCT04590274
Polyphenol-rich extracts	NCT04404218 NCT04410510 NCT04873349 NCT04680819 NCT04487964 NCT04967755
Polyphenol-rich extracts in combination with other bioactive components	NCT04400890 NCT04392141 NCT04382040 NCT04403646 NCT04810728 NCT04621149
Polyphenol-rich extracts in combination with drugs	NCT04374019 NCT04501965 NCT04530617
